# Secretor Status is Associated with Susceptibility to Disease in a Large GII.6 Norovirus Foodborne Outbreak

**DOI:** 10.1007/s12560-019-09410-3

**Published:** 2019-10-29

**Authors:** Sumit Sharma, Marie Hagbom, Beatrice Carlsson, Joanna Nederby Öhd, Mona Insulander, Ronnie Eriksson, Magnus Simonsson, Micael Widerström, Johan Nordgren

**Affiliations:** 1 Division of Molecular Virology, Department of Clinical and Experimental Medicine, Linköping University, Linköping, Sweden; 2 Department of Communicable Disease Control and Prevention, Stockholm County Council, Stockholm, Sweden; 3 Department of Medicine, Solna, Karolinska Institute, Stockholm, Sweden; 4 European Union Reference Laboratory (EURL) for Foodborne Viruses, National Food Agency, Uppsala, Sweden; 5 Department of Clinical Microbiology, Umeå University, Umeå, Sweden

**Keywords:** Norovirus, Outbreak, Host genetics, Histo-blood group antigens, GII.6

## Abstract

Norovirus is commonly associated with food and waterborne outbreaks. Genetic susceptibility to norovirus is largely dependent on presence of histo-blood group antigens (HBGA), specifically ABO, secretor, and Lewis phenotypes. The aim of the study was to determine the association between HBGAs to norovirus susceptibility during a large norovirus foodborne outbreak linked to genotype GII.6 in an office-based company in Stockholm, Sweden, 2015. A two-episode outbreak with symptoms of diarrhea and vomiting occurred in 2015. An online questionnaire was sent to all 1109 employees that had worked during the first outbreak episode. Food and water samples were collected from in-house restaurant and tested for bacterial and viral pathogens. In addition, fecal samples were collected from 8 employees that had diarrhea. To investigate genetic susceptibility during the outbreak, 98 saliva samples were analyzed for ABO, secretor, and Lewis phenotypes using ELISA. A total of 542 of 1109 (49%) employees reported gastrointestinal symptoms. All 8 fecal samples tested positive for GII norovirus, which was also detected in coleslaw collected from the in-house restaurant. Eating at the in-house restaurant was significantly associated with risk of symptom development. Nucleotide sequencing was successful for 5/8 fecal samples and all belonged to the GII.6 genotype. HBGA characterization showed a strong secretor association to norovirus-related symptoms (*P* = 0.014). No association between norovirus disease and ABO phenotypes was observed. The result of this study shows that non-secretors were significantly less likely to report symptoms in a large foodborne outbreak linked to the emerging GII.6 norovirus strain.

## Introduction

Norovirus is the most common cause of viral gastroenteritis in humans and is associated with 18% of diarrheal diseases and 200,000 deaths annually worldwide, mostly among children in developing countries (Lopman et al. 2016). Besides sporadic cases, norovirus is also the most common agent in outbreaks of gastroenteritis, due to its highly contagious nature and ease of spread through contaminated water and food. Globally, norovirus is estimated to be responsible for almost 50% of the foodborne outbreaks (Patel et al. 2009). A study involving 13 European countries reported 9430 norovirus foodborne outbreaks during 2002–2006 (Kroneman et al. 2008), with 78 of these being from Sweden (Kroneman et al. 2008). Norovirus-related outbreaks within the European Union have been mainly due to contaminated vegetables, bivalve mollusks, fruits, cereals, sprouts, herbs, and spices (EFSA and ECDC 2017, 2018). Each year, a large number of norovirus outbreaks are reported to occur globally in tourism (mainly cruises), sports, and hotel industry (restaurants), besides occasional outbreaks in hospitals and wards (Harris et al. 2010), leading to enormous economic loss.

Norovirus strains circulating worldwide are classified into 7 genogroups each having several genotypes. Genogroups I and II are commonly found to infect humans and both these genogroups have been found to cause outbreaks. A systematic review based on PCR confirmed human norovirus outbreaks during 1993–2011 reported that waterborne outbreaks were significantly associated with GI strains while GII strains mainly caused healthcare-related and winter outbreaks (Matthews et al. 2012). Within GII norovirus, genotype 4 (GII.4) strains account for most of the known cases of norovirus gastroenteritis and have been globally predominant over the last decades. Apart from GII.4, the GII.6 genotype has in recent years accounted for considerable proportion of norovirus cases, during some periods being the second most common genotype. Between 2005 and 2016, the annual GII.6 prevalence in Europe ranged between 2.0% and 10.3% of strains submitted to the NoroNet network (van Beek et al. 2018). Moreover, GII.6 strains accounted for an average 5.4% of norovirus outbreaks in USA reported to CaliciNet during 2013–2016, with a maximum of 10.3% in 2014–2015, making it the second most common genotype involved in outbreaks that year (Cannon et al. 2017).

Although humans of all ages can be infected with norovirus, not all individuals respond with diarrhea or vomiting. A previous study reported primary attack rate of 50% due to foodborne norovirus outbreaks (Matthews et al. 2012). This high but not complete attack rate could be due to several reasons including adaptive or innate factors. For instance, approximately 20% of Caucasian population is strongly resistant to GII.4 norovirus infections due to a nonsense mutation in the fucosyltransferase 2 (FUT2) gene (Nordgren et al. 2016). FUT2 adds a fucose to the precursor H type 1 antigen, which determines secretor status of an individual. Another fucosyltransferase, FUT3, adds a fucose to the precursor H type 1 antigen and/or the H antigen, making Lewis a or Lewis b antigen in non-secretors and secretors, respectively. These histo-blood group antigens (HBGA) are found in saliva and on cells in the gut epithelium and thus act as ligands to which norovirus binds to enter into the epithelial cells (Marionneau et al. 2002). Knowledge regarding genetic susceptibility to norovirus genotypes in humans can help understand their predominance in certain geographic regions and estimate attack rates during outbreaks. While the predominant GII.4 genotype has been shown to almost exclusively infect secretors, genotypes GII.2 and GI.3 can infect both secretors and non-secretors (Nordgren et al. 2016). A study from the USA (Currier et al. 2015), investigating secretor status and norovirus susceptibility, found that 95% of children infected with GII.6 were secretors, compared to 76% secretors in norovirus-negative controls, thus suggesting a strong secretor specificity for genotype GII.6. However, more remains to be elucidated on how the susceptibility to the second most common genotype GII.6 is dependent on histo-blood group glycans including secretor, but also Lewis and ABO. Therefore, the current study aimed to determine association of HBGA with norovirus susceptibility during a large foodborne outbreak linked to genotype GII.6 in an office-based company in Stockholm, Sweden in 2015.

## Methods

### Outbreak Description and Sample Collection

A large foodborne norovirus outbreak was reported in Sweden that occurred in two episodes during a three-week period in 2015 in a large office-based company in Stockholm. The Department of Communicable Disease Control and Prevention in Stockholm County (SmSt) and the Environmental Health Administration of Stockholm municipality (MoH) acted on the outbreak. Within three days from the outbreak onset, SmSt sent out an online questionnaire to all employees that had worked during the outbreak (*n* = 1109). Additionally, MoH performed a meticulous inspection of the in-house restaurant and collected samples of food (arugula, coriander, salad mix, and coleslaw) and water. Fecal samples of eight employees that had reported gastrointestinal (GI) symptoms were collected. The restaurant personnel were initially not included in the outbreak investigation, but the employer initiated sampling of 10 restaurant personnel for bacterial enteropathogens without consulting SmSt. On day 4, the restaurant was closed due to the outbreak.

Again, in the beginning of third week from day 1, SmSt was contacted by the company since an additional 54 individuals had fallen ill with GI-symptoms within a two-day onset period. Fecal samples from three employees with GI-symptoms and all 10-restaurant personnel were collected. Of these 10, one of the restaurant personnel also fell ill with GI-symptoms at the same time as the company employees. The MoH made a new inspection of the in-house restaurant and collected samples of iceberg lettuce to be sent for analysis.

### Bacterial and Virus Detection in Food, Water, and Fecal Samples

During the first outbreak episode, fecal samples of office employees were cultured for *Salmonella*, *Shigella*, *Campylobacter*, and *Yersinia* and analyzed for norovirus by qPCR GeneXpert (Cephid, Sunnyvale, United States), while fecal samples of restaurant personnel were only tested for *Salmonella*, *Shigella*, *Campylobacter*, and *Yersinia*. During the second outbreak the fecal samples of both office employees and restaurant personnel were tested for norovirus. The food and water samples from the first outbreak episode and iceberg lettuce from the second outbreak episode were tested by the National Food Agency according to ISO/CEN 15,216–2:2013.

### Nucleotide Sequencing of Norovirus-positive Samples

The partial nucleocapsid (NS) region of the viral protein 1 gene of five of the eight norovirus-positive fecal samples was amplified by PCR or nested PCR, followed by Sanger sequencing of the amplicons as described previously (Franck et al. 2014; Nordgren et al. 2010). ClustalW algorithm with default parameters in BioEdit software version 7.2.5 was used for multiple sequence alignment and phylogenetic trees were constructed with Maximum Likelihood algorithm using the Kimura 2 parameters model and gamma-distributed rate heterogeneity, with MEGA software, version 7. The topology of inferred phylogenetic tree was supported by bootstrapping using 1000 pseudoreplicates.

### Saliva Phenotyping

To investigate genetic susceptibility, saliva samples were collected for determining secretor, Lewis, and ABO phenotypes. A written informed consent for saliva phenotyping was obtained; the study, including consent procedure, was approved by the regional ethical board in Linköping (Dnr 2016/189–31). In total, saliva samples from 121 individuals having responded the questionnaire for the first outbreak episode were collected and successfully phenotyped. Secretor status was determined by the use of *Ulex europaeus* agglutinin (UEA-I), which recognizes Fucα1-2Gal-R present in saliva of secretors as described (Nordgren et al. 2014). Lewis and ABO phenotypes were determined by the use of monocloncal antibodies as described (Nordgren et al. 2014). Briefly, ELISA plates were coated with saliva, diluted 1:500 in coating buffer (0.1 M carbonate–bicarbonate buffer, pH 9.6); and incubated for 2 h at 37 °C followed by 4 °C overnight. The following day, the plates were incubated with antibodies α-A (Diagast, Loos France), α-B (Diagast), α-Lewis a (Seraclone, Bio-Rad, Solna, Sweden), and α-Lewis b (DiaClon, Bio-Rad) for 1.5 h at 37 °C, followed by incubation with HRP-conjugated goat anti-mouse IgG (Bio-Rad) for 1.5 h at 37 °C. For the UEA-I lectin assay, after blocking 1 h at 37 °C with 3% BSA, HRP-conjugated *Ulex europaeus* agglutinin (UEA-I, Sigma Aldrich, Sweden) diluted 1:3200 in PBS (starting conc. 1mg/ml) was added and incubated for 1.5 h at 37 °C. The reactions were developed using TMB (Fisher Scientific, Lund, Sweden) and stopped by addition of 2M H_2_SO_4_. Previously geno- and phenotyped secretor-positive, secretor-negative, Lewis-positive (Lewis a and b), and Lewis-negative saliva samples were used as controls in each plate. Individuals who did not answer any of the 3 questions pertaining to sickness, diarrhea, or vomiting in the initial online questionnaire were excluded in analysis (*n* = 11). To further ensure stringent inclusion criteria, persons who had answered yes for sickness but answered “no” to both vomiting and diarrhea were also excluded from analysis (*n* = 12).

### Statistical Analyses

To determine the association of HBGA in saliva to norovirus-associated symptoms, Fisher’s exact test with two-tailed significance was performed using SPSS version 24.

## Results

**Fig. 1:**
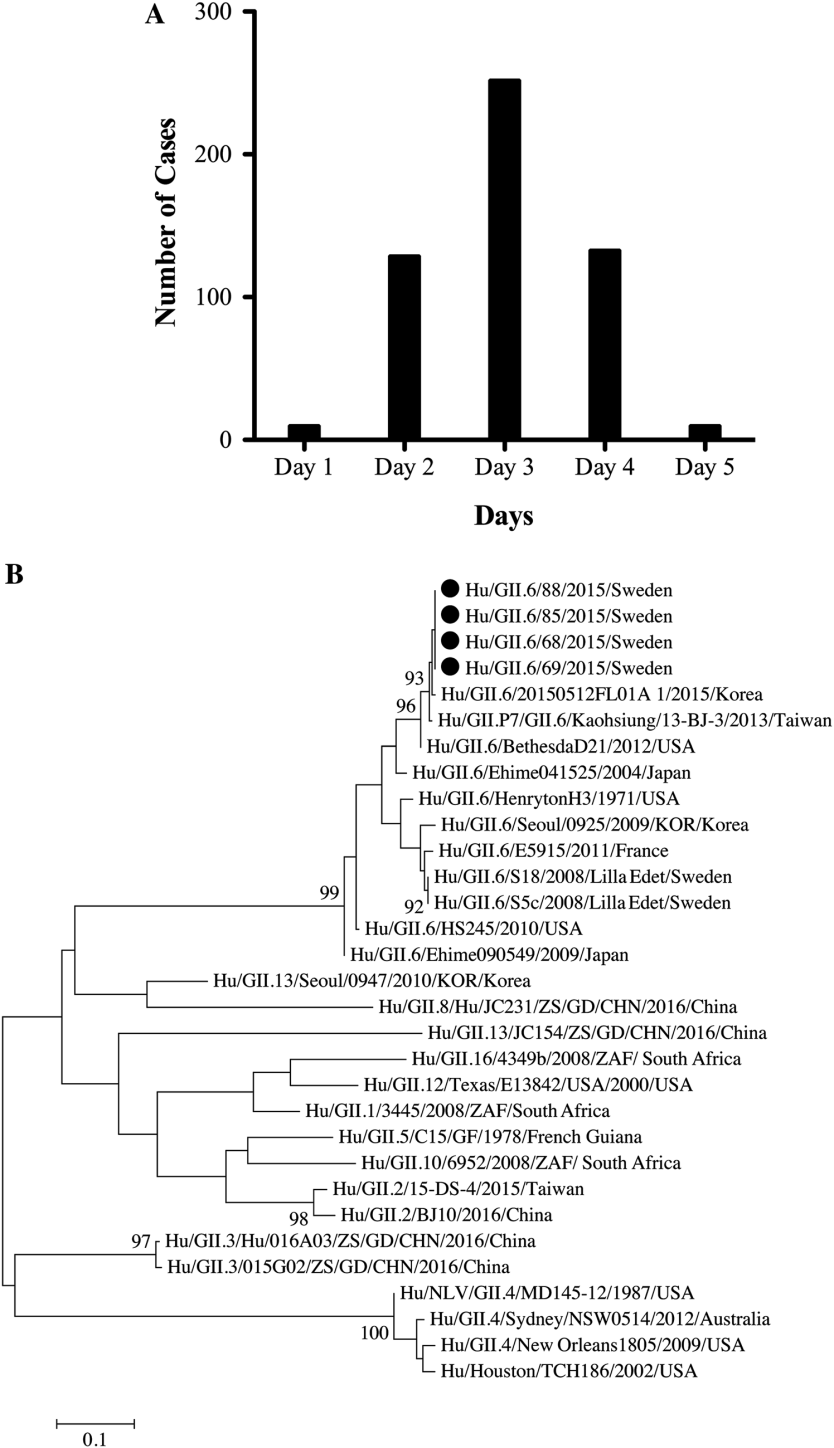
**a** Number of cases reported with norovirus-related illness (*n* = 542) during the first episode of GII.6 foodborne outbreak in a large office-based company in Stockholm, Sweden; **b** Phylogenetic tree showing clustering of four GII.6 norovirus strains (shown as filled circle) detected during the outbreak with reference GII.6 norovirus strains. The tree was generated on nucleotide sequences of the NS region (nt 1–272, ORF2). The scale bar represents number of nucleotide substitutions per site, and bootstrap values ≥ 85 are shown. Accession numbers of the strains sequenced are MH550088 for strain Hu/GII.6/68/2015/Sweden, MH550089 for strain Hu/GII.6/69/2015/Sweden, MH550090 for strain Hu/GII.6/85/2015/Sweden, and MH550091 for strain Hu/GII.6/88/2015/Sweden

All employees that had worked at the office during the first outbreak episode (*n* = 1109) answered the online questionnaire. Epidemiological data from the questionnaire showed that 542 out of the 1109 employees (attack rate 49%) responded that they experienced GI-symptoms (nausea, vomiting, or diarrhea) within the first 5 days (Fig. 1a). The median age of those who reported GI-symptoms was 43 years (range 22–73), and 54.2% (294/542) were men. Most of the symptomatic individuals had eaten in the restaurant in close time to onset of symptoms. Eating at the in-house restaurant on either of the two first days of the outbreak gave a relative risk of five (95% CI 3.4–7.4), *P* value < 0.0001) to symptom development.

All 8 fecal samples from individuals with symptoms of vomiting and/or diarrhea during the first outbreak episode tested positive for GII norovirus and negative for *Salmonella*, *Shigella*, *Yersinia*, and *Campylobacter*. The asymptomatic restaurant personnel also tested negative for *Salmonella*, *Shigella*, *Yersinia*, and *Campylobacter*. Analysis of food items (coriander, arugula, salad mix, and coleslaw) clearly showed norovirus GII in the coleslaw but on low levels (Ct-value 37). *Enterobacteriaceae* and *Listeria monocytogenes* were found in indicated food items, with the levels of *Listeria monocytogenes* above the microbiological criteria for recommended consumption. The water samples were without remarks. During the second outbreak episode, three employees with GI-symptoms were tested for norovirus, all were positive for GII norovirus. Two of the 10 restaurant personnel also tested positive for GII norovirus, including one symptomatic.

Amplification of the partial viral protein 1 gene and nucleotide sequencing was successful for five of the eight available samples from the first outbreak episode, with one sample yielding short read (130 bp) demonstrating genotype GII.6 but not used in subsequent phylogenetic analysis. The four sequenced norovirus strains had 100% nucleotide identity among each other and belonged to GII.6 genotype (Fig. 1b). The outbreak strains showed highest nucleotide identity (99.6%) with 20150512FL01A_1 strain detected in South Korea. Further, these strains shared only 90.5% nucleotide identity with S18 and S5C GII.6 strains that were isolated in Sweden during a waterborne norovirus outbreak in 2008 (Nenonen et al. 2012). Attempts to genotype the GII norovirus detected in the coleslaw were, however, unsuccessful, likely due to low viral load.

To investigate genetic susceptibility to disease during the outbreak, saliva samples were analyzed for individuals affected during the first outbreak episode. Of the 98 included saliva samples, 86 (87.8%) and 12 (12.2%) were phenotyped as secretors and non-secretors, respectively, while 72 (73.5%), 10 (10.2%), and 16 (16.3%) were Lewis b, Lewis a, and Lewis negatives, respectively. Among the secretors, 56% (*n* = 48/86) reported vomiting and/or diarrhea compared to 17% (*n* = 2/12) of the non-secretors (*P* < 0.05, Table 1). Among Lewis b positives, 56% (*n* = 40/72) reported diarrhea and/or vomiting, compared to only 10% (*n* = 1/10) of Lewis a phenotype. Among secretor positives, none of the phenotypes (A, B, AB, or O) showed any association to norovirus-related symptoms (Table 1). Overall, norovirus-associated symptoms were reported from individuals of all secretor, Lewis, or ABO phenotypes status, however, with a significantly lower proportion of non-secretors or individuals with the Lewis a phenotype.

**Table 1:** Distribution of Lewis, secretor, and ABO phenotypes in individuals with or without symptoms during the norovirus outbreak

Saliva phenotype	Total number	Symptoms	No symptoms	*P* value^1^
		n (%)	n (%)	
Lewis phenotype				
Lewis a	10	1 (10)	9 (90)	0.007
Lewis b	72	40 (56)	32 (44)	0.17
Lewis negative	16	9 (56)	7 (44)	0.79
ABO phenotype^2^				
A	32	17 (53)	15 (47)	0.82
B	13	8 (61)	5 (39)	0.77
AB	1	1 (100)	0 (0)	N.A
O	40	22 (55)	18 (45)	1.0
Secretor status^3^				
Positive	86	48 (56)	38 (44)	0.014
Negative	12	2 (17)	10 (83)	0.014

^1^Fisher’s exact test with two-tailed significance

^2^Only analyzed for secretor-positive individuals

^3^Three saliva samples negative for α1,2 fucose (present in secretors) using UEA-1 agglutinin assay and negative for blood group antigens, exhibited small amounts of Lewis b in addition to Lewis a. These samples were classified as non-secretors

## Discussion

This reported outbreak occurred in two episodes with the first outbreak episode having strong correlation with eating at the in-house restaurant as indicated by the abrupt initiation and fast spread as well as the relative risk linking symptoms to eating in the in-house restaurant. The second outbreak episode had similar characteristics and was in addition to food, linked to restaurant personnel and employees. Notably, the restaurant personnel also received daily meals from the restaurant kitchen. In this two-episode outbreak, both the infectious agent and location were identified early in the process; however, the initial source could not be discerned. Nevertheless, a plausible explanation for the outbreak would be norovirus GII-contaminated coleslaw. The first episode had an epidemiology consistent with a foodborne outbreak with a clear predominance of norovirus GII.6 infections. The second episode is less clear and was probably a mixture of foodborne and person-to-person transmissions. The close connection between the two episodes indicates spread of the same norovirus genotype, which could unfortunately not be confirmed due to unavailability of fecal samples for genotyping in the second outbreak episode. Attempt to genotype the norovirus GII-positive coleslaw sample was made, but was unsuccessful due to low concentrations of the virus, as indicated by high Ct values, a common issue regarding norovirus in food samples (Bosch et al. 2018).

The clinical importance of the emerging GII.6 norovirus should not be underestimated; it was the second most common genotype in 2014–2015 outbreaks in the USA (Cannon et al. 2017) and the second most common genotype (14%) in young children in USA (Currier et al. 2015). In this outbreak investigation, individuals with non-secretor and/or Lewis a phenotypes were significantly less likely to report norovirus-associated symptoms during the described outbreak compared to secretors, although a few non-secretors (*n* = 2) and Lewis a positives (*n* = 1) reported symptoms. This is in agreement with a study from the USA, where 95% of children infected with GII.6 were secretors (Currier et al. 2015), compared to 76% secretor prevalence in the norovirus-negative healthy controls, thus showing a strong but not complete secretor specificity for genotype GII.6. It is possible that non-secretors are only partially protected to infections with GII.6, possibly due to weak interactions between HBGAs in non-secretors and GII.6 norovirus. A previous in vitro study further observed strong binding of a GII.6 virus-like particle (VLP) with the Lewis b carbohydrate as well as secretor-positive saliva, whereas the Lewis a antigen and non-secretor saliva demonstrated weak binding (Shirato et al. 2008). The same study (Shirato et al. 2008), however, used VLPs from two different GII.6 strains, with the other GII.6 VLP exhibited a different binding profile, e.g., with no binding to non-secretor saliva. This shows that there might be differences even between strains of the same genotype and highlights the importance for in vivo studies to elucidate genetic susceptibility for specific norovirus strains.

The proportion of secretors in this study was higher than previously reported from Sweden (Nordgren et al. 2016). This may be due to the fact that individuals with symptoms during the outbreak were more likely to provide saliva samples.

Similar proportions of secretor individuals with each blood type (A, B, and O) experienced norovirus-related symptoms during the outbreak period. The only individual in this study with blood type AB also reported symptoms. This is in agreement with studies reporting GII.6 VLP binding to saliva samples of all ABO phenotypes (A, B, AB, and O) (Zheng et al. 2017; Shirato et al. 2008). Stratifying by secretor status, we found no effect of the Lewis antigen, with Lewis-positive and Lewis-negative secretors being equally represented among persons with symptoms (data not shown). The number of Lewis-negative non-secretors was too small for reliable analysis (*n* = 2). However, one Lewis-negative non-secretor and one Lewis-positive non-secretor reported norovirus-associated symptoms.

This study has several limitations. We only had consent for saliva phenotyping and not genotyping, which is a complementary test for determining secretor status. The majority of samples, however, showed matched results between the ABO, Lewis, and secretors phenotype assays, e.g., saliva negative for α1,2 fucose (non-secretors) were also negative for Lewis b and blood group antigens. Three saliva samples, however, that were negative for α1,2 fucose using the UEA-1 agglutinin assay and negative for blood group antigens, had small amount of Lewis b in addition to Lewis a antigen. The reason for this is unclear but may constitute a weak secretor phenotype, i.e., reduced but not completely inactivated FUT2 enzyme activity (Ferrer-Admetlla et al. 2009). As genotyping was not possible in this study, and weak secretors have a similar susceptibility pattern to norovirus as non-secretors (Liu et al. 2014), these samples were classified as non-secretors due to the negativity for α1,2 fucose. Previous studies have observed a strong correlation by non-secretor genotype and non-secretor phenotype by the UEA-1 agglutinin assay (Bucardo et al. 2018). Another limitation is that only five out of the eight available feces samples were successfully genotyped and the norovirus-positive coleslaw could not be genotyped. According to guidelines at SmSt, if norovirus is detected after primary sampling and further corroborated through epidemiology and interview/questionnaires, no further samples are taken. This is why a relatively low number of fecal samples were collected relative to the number of individuals affected. However, all sequenced samples belonged to the same genotype and exhibited a 100% nucleotide identity, strongly indicating a single norovirus genotype causing the outbreak, although infection with other norovirus genotypes cannot be ruled out.

## Conclusion

The present study investigated genetic susceptibility to disease in a large foodborne norovirus outbreak linked to genotype GII.6 with an attack rate of 49%. Our results show that coleslaw was contaminated and that that non-secretors were significantly less likely to report symptoms.
